# Early-career registered nurses’ work experience and nurse outcomes in South African hospitals: a cross-sectional survey

**DOI:** 10.1186/s12912-025-03188-5

**Published:** 2025-05-13

**Authors:** Nicholin Scheepers, Siedine Coetzee, Erika Fourie

**Affiliations:** 1 School of Nursing Science, Faculty of Health Sciences, North-West University, Private Bag X1290, Potchefstroom, 2520 South Africa; 2 Department of Statistical Consultation, Faculty of Humanities, North-West University, Private Bag X1290, Potchefstroom, 2520 South Africa

**Keywords:** Nurse outcomes, Early-career registered nurses, Job satisfaction, Career satisfaction, Workplace violence, Emotional exhaustion, Job turnover, Career turnover

## Abstract

**Background:**

Early-career registered nurses within their first five years of practice, experience the highest turnover rates in the nursing profession, with 20 to 40% leaving their jobs within the first two years. These high turnover rates are due to poor nurse outcomes. Despite growing global research on ECRNs, limited evidence exists within the South African context. This study aimed to examine the nurse outcomes of ECRNs in both private and public hospitals of South Africa and to explore the association between their work experience and these outcomes.

**Methods:**

Total population sampling was used to recruit ECRNs in private and public hospitals of South Africa. The cross-sectional survey instrument measured job and career satisfaction, workplace violence, emotional exhaustion (burnout), and intent to leave job and career. Descriptive statistics and ANOVA-type hierarchical linear modelling were used to present the differences in ECRNs’ work experience and their nurse outcomes.

**Results:**

Dissatisfaction rates among ECRNs were notable, with 34.9% dissatisfied with their jobs and 23.1% dissatisfied with their career choice. Workplace violence, including personal (M = 1.60; SD = 0.78) and physical (M = 1.44; SD 0.73) violence, was reported, primarily from managers and supervisors. Emotional exhaustion was prevalent (M = 30.85; SD = 14.62), with 38.9% of the respondents intending to leave their job and 14.5% considering leaving the profession. NGs experienced more workplace violence and were least satisfied with professional status and advancement opportunities, although most satisfied with their co-workers. First-year ECRNs were most satisfied with independence and recognition, while fifth-year ECRNs were least dissatisfied with educational opportunities. Private sector ECRNs reported higher job satisfaction, work-life balance, salary, recognition, educational opportunities, work schedule, and advancement prospects than those in the public sector.

**Conclusion:**

The study highlighted poor nurse outcomes among ECRNs, especially regarding emotional exhaustion, job satisfaction, and intent to leave. NGs face significant workplace violence and dissatisfaction with advancement opportunities. Urgent interventions are needed, such as transition-to-practice programmes with mentors, fostering positive work environments, and supportive leadership, to enhance ECRNs’ quality of work life and reduce attrition rates in South Africa’s health sector.

## Introduction

Nurse shortages are escalating globally, driven by the increasing retirement of experienced nurses and the growing trend of new nurse graduates (NGs) planning to leave their positions within their first year of employment [1, 2]. A significant contributor to nurse shortage in South Africa is the retirement of experienced nurses, which is due to the scarcity of replacements [3]. South Africa faces the demographic challenge of 27% of its registered nurses being in their 50s, 26% in their 40s, and only 21% in their 30s [4].

There are discrepancies in the supply of nurses to maintain adequate number of nursing workforce in response to an ageing population, retirement, as well as recruitment and retention of existing nurses, making it difficult to respond to the growing demand [5, 6]. These shortages further exacerbate staffing challenges, compromising the quality of patient care and increased job dissatisfaction and a high turnover rate among early-career registered nurses (ECRNs). Therefore, it is imperative to focus on retaining ECRNs as a crucial strategy for ensuring a sustainable future supply of nursing professionals.

## Literature review

### Job satisfaction and career commitment

Early-career registered nurses (ECRNs), including newly graduated nurses (NGs), are defined as those within their first five years of professional practice [7]. Research consistently shows that ECRNs; particularly within the first two years face alarmingly high turnover rates of 20–40% [8]. These rates are driven by poor nurse outcomes, which include low job satisfaction, strained workplace relationships, compromised health and wellbeing, and reduced productivity and effectiveness [9, 10].

Job satisfaction is a critical component of nurse outcomes and a strong predictor of retention. It refers to nurses’ positive evaluations of their work environment based on how well it meets their expectations and needs [11]. High dissatisfaction rates have been reported globally in Japan (66%), China (55%), South Korea (50%), and the UK (42%) [12]. In the USA, dissatisfaction among ECRNs typically peaks between 6 and 18 months, often linked to patient care demands, work schedules, salaries, and limited educational opportunities [13]. The first 6 months post-graduation are often the most stressful, aligning with the highest levels of job dissatisfaction. Interventions such as preceptorships, mentorships, and residency programmes have proven effective in mitigating this trend [14].

In South Africa, job dissatisfaction affects 32.2% of nurses, with 46.1% intending to leave their current workplaces. Notably, public sector nurses (54%) report significantly more dissatisfaction than their private sector counterparts [14–16]. However, there is limited data specifically focusing on ECRNs in South Africa and other low- and middle-income countries (LMICs).

### Intention to leave and turnover

Job dissatisfaction is a significant factor contributing to the intention to leave a job. Studies have reported that the intention of NGs to leave their jobs ranges from 16 to 57%. This trend was observed in studies conducted among NGs intending to leave their jobs in South Korea (16%), Canada (18–30%), and Turkey (42.5%) within their first year of employment. Additionally, between 37% and 57% expressed an intent to leave the nursing profession in their second year of employment [2, 18].

A systematic review conducted in sub-Saharan Africa regarding nurses’ intention to leave their jobs showed that 51.96% expressed an intention to leave. The highest intention to leave among nurses was observed in East Africa (58.03%), whereas South African nurses showed the lowest intention to leave their jobs (33.04%) [19]. Although these studies included all categories of nurses, it is apparent that there is a substantial difference between developed and developing countries in terms of experienced nurses’ and NGs’ intention to leave. De Oliveira et al. [20] have highlighted key predictors of high turnover rates and intention to leave as heavy workloads, economic instability, lack of recognition, and limited opportunities for professional advancement especially in the public sectors of developing countries.

### Burnout and mental well-being

Burnout, as defined by Maslach and Leiter [21], refers to a state of persistent physical, mental, or emotional exhaustion, it is a prevalent and severe challenge for ECRNs which significantly impairs retention and performance. Approximately 25–33% of healthcare workers report burnout symptoms [22, 23]. Among ECRNs, burnout often serves as a primary factor in decisions to exit the profession within their initial 5 years of practice [24].

A systematic review including 34 quantitative studies revealed that burnout among NGs was moderately high, primarily stemming from emotional exhaustion and stress. Interestingly, this burnout tended to decrease as job satisfaction improves over time [25]. Unpredictable and high-stress work environments, varying work schedule, work and time pressure, and lack of social support increases burnout risks [26]. Additionally, a Canadian longitudinal study revealed that burnout increases the risk of post-traumatic stress disorder in NGs, highlighting the importance of a positive work environment and supportive leadership for emotional resilience [27]. Although burnout rates in developed countries range between 25% and 33%, there is a significant lack of literature focusing specifically on ECRNs in South Africa. Existing studies report burnout prevalence in South Africa between 34.6% and 45.8% across both public and private healthcare, which is higher than developing countries rates [15, 28].

### Workplace violence

Workplace violence encompassing verbal, physical and emotional abuse, significantly impacts ECRNs morale safety and retention [29]. Globally, workplace violence against ECRNs has been widely reported. In the USA, 80% of ECRNs have experienced violence from patients or their families. In South Korea, 59.6% reported verbal abuse, along with threats (36.9%), physical violence (27.6%), bullying (25.6%), and sexual harassment (22.4%) [30].

Weaver [31] reported that ECRNs lack experience in the work environment, which makes them vulnerable to becoming victims of verbal abuse at work. Furthermore, due to the hierarchy in the workplace ECRNs are more prone to be disrespected by colleagues in higher positions on the hierarchy [32]. In South Africa a prevalence of 54–100% of workplace violence has been reported among nurses, with patients and their relatives being common perpetrators [33, 34]. A study conducted in KwaZulu-Natal in South Africa indicated that most nurses experienced higher incidences of personal workplace violence (M = 1.61; SD = 0.75) then of physical workplace violence (M = 1.43; SD = 0.69) [35].

### Strategies to improve nurse outcomes of ECRNs

To improve ECRNs outcomes and reduce turnover rates, several evidence-based strategies have been identified. Supportive leadership, where leaders are approachable, empathetic and empowering create positive work environmental and reduce stress and burnout [36]. ECRNs need continuous professional development through having access to education, training and career advancement opportunities, which will increase their job satisfaction and retention [37]. Competitive salaries, bonusses and recognition programmes have shown to be effective retention strategies, which need to be implemented in the public sectors of South African hospitals [38]. Additionally structured support programmes like, residency programmes, preceptorships and mentorship models, significantly lower turnover by enhancing confidence, clinical skills acquisition and workplace integration [37].

Despite the vast literature available on nurse outcomes worldwide, previous studies focuses on the broader nursing population and most studies in high-income studies focus on ECRNs outcomes. There is an existing evidence-gap in South African literature pertaining to ECRNs outcomes, who face unique challenges that require context-specific interventions.

## Aim of the study

This study aimed to examine the nurse outcomes of ECRNs in a South African context, and the relationship between their work experience and these outcomes. This is the first study to be conducted nationally with a specific focus on ECRNs, providing valuable insights into the unique challenges and experiences faced by this group of nurses.

## Methods

### Study design

This study used a cross-sectional descriptive design. The study and its reporting were guided by the Strengthening the reporting of observational studies in epidemiology (STROBE) checklist of items that have be included in reports of cross-sectional studies. Adhering to the STROBE checklist helps researchers present their finding transparently, facilitating critical appraisal and reproducibility of the research.

### Sample and setting

The study formed part of a larger study (NWU-00033-19-A1) which was conducted in all nine provinces of South Africa and included four major private hospital groups with 116 hospitals. For each province, the public sector hospitals included the largest central hospital (*n* = 5), and in those provinces with no central hospital, the largest tertiary hospital was selected (*n* = 4). Then the regional and district hospitals in closest proximity to the selected central/tertiary hospital were selected. District hospitals were stratified into small (*n* = 4), medium (*n* = 3), and large (*n* = 2), according to National Department of Health regulations relating to categories of hospitals [39]. A total of 27 hospitals were included in the sample.

In the larger study, the researchers used, total sampling in both sectors to select *n* = 4554 nurses, which included registered nurses/midwives, NGs, enrolled nurses and midwives and auxiliary nurses. The nursing staff had to have been working in the hospitals for at least 3 months, with student nurses excluded.

This study is a subset study for the larger study where the total population of 359 ECRNs were purposively selected. These ECRNs were included based on the definition that ECRNs are nurses with 3 months to 5 years of working experience. As confirmation a sample size calculation was performed in g-power using the F-tests as the Test Family and the ANOVA: fixed effects, special, main effects and interactions as the statistical test to take the structure of the data into account. The parameters were specified as follow: Effect size f as large (0.4) and medium (0.25), α err prob as 0.05, Power (1-β err prob) as 0.95, Numerator df as 10, Number of groups 6. The total sample sizes calculated were 162 and 400, which is in line with the realised sample size of 359.

### Data collection

Data collection commenced in April 2021 and continued to June 2022 for both health sectors. The hospital Chief Executive Officers (CEOs) of hospitals provided goodwill consent as gatekeepers, while the Nursing Service Managers acted as mediators. They identified an independent clinical co-ordinator from the clinical training units to assist with the recruitment of nurses and the process of obtaining informed consent. The clinical coordinator received training from the researchers to carry out these tasks.

Participants had 24 h to decide whether they wanted to take part in the study; once informed consent was signed, the independent person gave the participants an envelope containing the self-administered survey, to complete within two days. After completion of the survey, participants were provided with a post box at the clinical training units for submitting the surveys. For private hospitals entrance to the facilities occurred in a manner similar to that at the public hospitals; however, participants completed the informed consent and survey online using SurveyMonkey. Surveys were completed anonymously, but participants were asked to identify the ward and hospital, for data analysis purposes.

### Questionnaire

The self-report instrument measured job and career satisfaction using the RN4CAST job and career satisfaction scale (12 items). This instrument was used as single items in RN4CAST studies conducted in Europe and the USA [40], and in a South African study [15]. The Workplace Relationships Negative Acts questionnaire measured bullying and victimisation (15 items) and was validated by Einarsen and Notelaers [41] with Cronbach’s alphas measured for physically intimidating bullying (Cronbach’s alpha = 0.83) and personal-related bullying (Cronbach’s alpha = 0.96). The Maslach Burnout Inventory measured emotional exhaustion (9 items); the instrument demonstrated good validity and reliability, with a Cronbach’s alpha of 0.89 [15]. The RN4CAST job and career turnover intent measured intent to leave the workplace or the profession (2 items); the validity and reliability was confirmed by studies conducted in Europe, the USA and South Africa, as mentioned. Demographic characteristics that were measured were gender, age, nurse category, current unit, current employment status, and years worked as a registered nurse.

### Data analysis

Data were entered and analysed in IBM SPSS Statistics version 26 of 2014. Paper-based surveys were captured with an optical mark recognition scanner, while online surveys were imported to a Microsoft Excel spreadsheet. Descriptive statistics used were frequencies, percentages, means and standard deviations. ANOVA-type hierarchical linear modelling (HLM) was conducted to test for differences in ECRNs’ work experience NGs, first to fifth year of nursing experience [6 groups] and nurse outcomes, as the means of the work experience and not the regression coefficients were important in interpretation of the results. After the ANOVA-type HLM, pair- wise post hoc comparisons were done to determine the statistically significant differences between the groups. The authors did not focus on regression coefficients (Beta), as they were interested in how the means of the different groups differed statistically. The analysis focused on how the groups (work experience) differed practically, therefore the focus was on effect size, although statistical significance is also reported. Normality of the data was tested using the Kolmogorov-Smirnov test, but due to the unlikelihood of non-significant p-values in such a large sample size, more significance was ascribed to results from Q-Q plots. The points in the Q-Q plot must lie close to the straight line to retain the assumption that the data distribution is normal for all variables [42].

### Ethical considerations

Ethical approval of this study was obtained from the Faculty of Health Research Ethics Committee (NWU-00033-19-A1). Confidentiality of participants’ information was maintained through confidentiality agreements between the statistician and researchers. Anonymity of both paper-based surveys and online-surveys were maintained by not requiring personal information of participants. Informed consent was obtained from participants, which ensured voluntary participation. Online- surveys were downloaded and stored into an electronic file in a password protected computer, which the primary investigator (PI) of the larger study had access to. Hard copies data (paper-based surveys and informed consents) were locked in cupboard in the PIs office, which the PI only had access to.

## Results

### Demographics of participants

Participants were aged between 21 and 54 years, with an average age of 30 years (SD 5.951). The majority were female (81.6%), with a diploma in nursing (59.1%). The majority of ECRNs had 5 years working experience (28.4%), with the minority having I year work experience (11.1%) and 12.8% were NGs. Of the participants, 284 (79.1%) were permanently employed, with the majority working in surgical (*n* = 70; 19.5%), medical (*n* = 65; 18.1%) and critical care (*n* = 65; 18.1%) units. Most participants were working in the public sector (62.4%) and resided in Gauteng province (25.6%) (Table 1).

**Table 1:** Demographics of early-career registered nurses (*n* = 359)

Variable	*N* (%)
Age (yrs.)	359
Mean (SD)	30.08 (5.951)
Min–Max	21–54
**Gender**	
Female	293 (81.6)
Male	66 (18.4)
**Bachelor’s Degree in Nursing**	
Yes	140 (39.0)
No	212 (59.1)
*Missing value*	7 (1.9)
Nurse category	
Community Service Nurse (NGs)	46 (12.8)
Year 1 RN	40 (11.1)
Year 2 RN	54 (15)
Year 3 RN	54(15)
Year 4 RN	63 (17.5)
Year 5 RN	102 (28.4)
**Employment status**	
Permanently employed (full-time)	284 (79.1)
Permanently employed (part-time)	15 (4.2)
Fixed-term contract	8 (2.2)
Temporary/agency	50 (13.9)
*Missing value*	*2 (0.6)*
**Nurse category**	
Registered nurse/midwife	313 (87,2)
Nurse graduate	46 (12.8)
**Specialty area of current unit**	
Medical	65 (18.1)
Surgical	70 (19.5)
Trauma	32 (8.9)
Maternity	56 (15.6)
Critical care	65 (18.1)
Theatre	12 (3.3)
Pediatrics	25 (7)
Other	1 (0.3)
Neonatal intensive care unit	9 (2.5)
Management	1 (0.3)
Education	0 (0)
Psychiatry	10 (2.8)
Oncology	2 (0.6)
Burns	0 (0)
COVID-19	9 (2.5)
*Missing value*	2 (0.6)
**Public/private sector**	
Private	135 (37.6)
Public	224 (62.4)
**Province**	
Eastern Cape	46 (12.8)
Free State	20 (5.6)
Gauteng	92 (25.6)
KwaZulu-Natal	46 (12.8)
Limpopo	24 (6.7)
Mpumalanga	18 (5)
North-West	42 (11.7)
Northern Cape	14 (3.9)
Western Cape	56 (15.6)
*Missing value*	1 (0.3)

Nurse outcomes of ECRNs are presented as job and career satisfaction (Table 2), workplace violence and burnout (emotional exhaustion) (Table 3), and intent to leave job and career (Table 4).

### Job and career satisfaction

Of the ECRNs, 125 (34.9%) were dissatisfied with their jobs and 83 (23.1%) were dissatisfied with their career choice. The aspects of the job that they were most dissatisfied with included appreciation and recognition (71.3%; *n* = 256), salary (71.0%; *n* = 255), and education opportunities (60.7%; *n* = 218), as stipulated in Table 2.

**Table 2:** Job and career satisfaction of early-career registered nurses in South Africa

Job and career satisfaction						
**Variable**	**M (SD)**	**(1)** **Very dissatisfied**	**(2)** **A little dissatisfied**	**(3)** **Moderately satisfied**	**(4)** **Very satisfied**	**Missing value**
**Job satisfaction**						
Overall job satisfaction	2.58 (0.898)	16.2% (58)	18.7% (67)	50.1% (180)	10.6% (38)	4.5% (16)
Work schedule	2.89 (0.923)	10.3% (37)	15% (54)	44% (158)	25.1% (90)	5.6% (20)
Opportunities for advancement	2.05 (1.006)	36.8% (132)	24.8% (89)	24% (86)	8.6% (31)	5.8% (21)
Independence at work	2.87 (0.870)	8.6% (31)	16.4% (59)	47.6% (171)	21.7% (78)	5.6% (20)
Professional status	2.89 (0.870)	8.6% (31)	15.6% (56)	47.9% (172)	22.3% (80)	5.6% (20)
Salary/wages	1.79 (0.941)	49.3% (177)	21.7% (78)	89.9% (68)	5.0% (18)	5.0% (18)
Educational opportunities	2.09 (1.024)	34.8% (125)	25.9% (93)	22.8% (82)	10.3% (37)	6.1% (22)
Appreciation, recognition, and rewards	1.83 (0.921)	44.6% (160)	26.7% (96)	18.1% (65)	5% (18)	5.6% (20)
Immediate supervisor/manager	2.74 (0.989)	15% (54)	16.2% (58)	40.9% (147)	21.4% (77)	6.4% (23)
Co-workers	2.94 (0.827)	7% (25)	14.2% (51)	50.7% (182)	22.8% (82)	5.3% (19)
**Career satisfaction**						
Career satisfaction		8.9% (32)	14.2% (51)	30.4% (109)	42.1% (151)	4.5% (16)

### Workplace violence and intent to leave

ECRNs experienced personal (M = 1.60; SD = 0.78) and physical (M = 1.44; SD = 0.73) workplace violence now and then, and the most common perpetrators were managers and supervisors (38.4%; *n* = 86). They experienced high levels of emotional exhaustion (M = 30.85; SD = 14.62) (Table 3). A total of 38.9% (*n* = 136) intended to leave their place of employment, and of these, 14.5% (*n* = 20) intended to leave the profession altogether (Table 4).

**Table 3:** Workplace violence and burnout of early-career registered nurses

Workplace violence			
Personal violence	1.60	0.78	1 (Never) – 5 (Daily)
Physical violence	1.44	0.73	1 (Never) – 5 (Daily)
**Variable**	**F (%)**		
**Perpetrators**			
Physicians	63 (17.5%)		
Nursing colleagues	75 (20.9%)		
Supervisors/managers	86 (24.0%)		
**Burnout**			
Emotional exhaustion	30.85	14,62	0 (Never) – 6 (Every Day)

**Table 4:** Early-career registered nurses’ intention to leave

Variable	Intention to leave	
	Frequency	%
**Do you plan on leaving your job in the next year?**		
Yes	136	38.9
No	214	61.1
**If yes**,** what type of work would you seek?**		
Nursing in another hospital	64	46.4
Nursing, but not in a hospital	54	39.1
Non-nursing career	20	14.5

### Association between demographics and nurse outcomes

T-tests and ANOVA’s were conducted as initial screening to assesses if there are any differences between the demographical groups (Table 1) with regards to nurse outcomes (Tables 2, 3 and 4). The private/public sector was the only demographic factor with significant differences s; specifically work schedule (p 0.007; d = 0.44), opportunities for advancement (*p* < 0.001; d = 0.86), salary (p 0.039; d = 0.41), educational opportunities (*p* < 0.001; d = 0.90), appreciation (*p* < 0.001; d = 0.82) and work-life balance (*p* < 0.001; d = 0.64) Therefore only this variable was considered in the further analyses.

### Comparison of work experience to nurse outcomes

ANOVA-type HLM was conducted to compare the ECRNs’ years of work experience (NGs, first to fifth year of nursing experience [6 groups]) to nurse outcomes (Table 5). Data are presented as means, covariance estimates parameters, statistical significance (p-value) and effect sizes (Cohen’s d). Researchers reported on medium and large effect size only; according to Cohen [43] 0.5 indicates a medium effect and 0.8 a large effect.

The largest effect size was reported for personal workplace violence (M = 2.34) of NGs in comparison to ECRNs with 1 year of experience (M = 1.38; *p* = 0.238; d = 1.25), 2 years of experience (M = 1.56; *p* = 0.238; d = 1.02), 3 years of experience (M = 1.66; *p* = 0.238; d = 0.88), 4 years of experience (M = 1.52; *p* = 0.238; d = 1.07) and 5 years of experience (M = 1.65; *p* = 0.238; d = 0.89). This means that NGs experience more frequent exposure to personal workplace violence than any other year group.

Large effect sizes were reported with regard to satisfaction with professional status for NGs (M = 2.38) in comparison to ECRNs with 1 year of experience (M = 3.02; *p* = 0.279; d = 0.76), 2 years of experience (M = 3.06; *p* = 0.279; d = 0.80), 3 years of experience (M = 3.05; *p* = 0.279; d = 0.79), and 4 years of experience (M = 2.99; *p* = 0.279; d = 0.72), and a medium effect size with 5 years of experience (M = 2.85; *p* = 0.279; d = 0.56). This means that NGs experience less satisfaction with professional status than any other year group.

There are medium to large effects reported for satisfaction with opportunities for advancement when comparing NGs (M = 1.71) and ECRNs with 1 year of experience (M = 2.23; *p* = 0.237; d = 0.57;), 2 years of experience (M = 2.08; *p* = 0.237; d = 0.41), and 4 years of experience (M = 2.32; *p* = 0.237; d = 0.68). This means that NGs experienced less satisfaction regarding opportunities for advancement than the other year groups.

There was a medium effect between NGs’ (M = 2.73) satisfaction with independence at work in comparison to ECRNs with 1 year of experience (M = 3.14; *p* = 0.410; d = 0.48), meaning that only in their first year of practice did ECRNs experience satisfaction with their independence at work.

Medium effects were reported for satisfaction with educational opportunities between NGs (M = 2.73) compared to ECRNs with 3 years of experience (M = 2.36; *p* = 0.627; d = 0.41) and 5 years of experience (M = 2.21; *p* = 0.627; d = 0.57), meaning that NGs felt more satisfied with the educational opportunities available. When looking at the ECRNs, there were medium effects between satisfaction with educational opportunities of first-year ECRNs (M = 2.67) and those of fifth-year ECRNs (M = 2.21; *p* < 0.001; d = 0.627), meaning that fifth-year ECRNs were the least dissatisfied with educational opportunities.

There was a medium effect between satisfaction with appreciation, recognition, and rewards of NGs (M = 1.87) and that of RNs in their first year (M = 2.22; *p* = 0.021; *p* < 0.0001; d = 0.42), meaning that only in their first year of practice did ECRNs experience satisfaction with appreciation, recognition, and rewards. There were medium effects between satisfaction with appreciation, recognition and rewards for ECRNs in their first year (M = 2.22) compared to ECRNs in their second year (M = 1.67; *p* = 0.021; d = 0.64), and fifth year (M = 1.76; *p* = 0.021; d = 0.54), as well as for fourth-year ECRNs (M = 2.11) compared to ECRNs in their second year (M = 1.67; *p* = 0.021; d = 0.52) and fifth year (M = 1.76; *p* = 0.021; d = 0.41), meaning that first-year ECRNs were most satisfied with appreciation, recognition and rewards out of all the ECRN groups.

There was also a medium effect between satisfaction with coworkers (M = 3.25) compared to ECRNs with 2 years of experience (M = 2.87; *p* = 0.102; d = 0.46), 3 years of experience (M = 2.83; *p* = 0.102; d = 0.51) and 5 years of experience (M = 2.87; *p* = 0.102; d = 0.46;). This means that NGs, and first- and fourth-year ECRNs were most satisfied with their coworkers. There was a medium effect between satisfaction with co-workers (M = 3.21) among ECRNs in year 4, compared to those in year 2 (M = 2.87; *p* = 0.102; d = 0.42), year 3 (M = 2.83; *p* = 0.102; d = 0.47) and year 5 (M = 2.87; *p* = 0.102; d = 0.42), meaning that fourth-year ECRNs were more satisfied.

Medium effects were reported for experience of physical workplace violence between NGs (M = 1.83) compared to ECRNs with 1 year of experience (M = 1.33; *p* = 0.845; d = 0.67), 2 years of experience (M = 1.47; *p* = 0.845; d = 0.49), 3 years of experience (M = 1.45; *p* = 0.845; d = 0.51), 4 years of experience (M = 1.42; *p* = 0.845; d = 0.56), and 5 years of experience (M = 1.49; *p* = 0.845; d = 0.45), meaning the NGs experienced physical workplace violence more often than the other groups. There was a medium effect between the emotional exhaustion of NGs (M = 40.01) and ECRNs with 2 years of experience (M = 33.17; *p* = 0.845; d = 0.47), meaning that ECRNs in their second-year experienced emotional exhaustion less often than NGs.

**Table 5:** Higher linear modelling of nurse outcomes of early-career registered nurses

Item	Work experience	Mean	Var		p-value	Effect sizes				
			MSE	Hosp		NG with	Year 1 with	Year 2 with	Year 3 with	Year 4 with
Intention to leave	New Graduate	0.43	0.2168	0.0244	0.706					
	Year 1	0.48				0.11				
	Year 2	0.35				0.17	0.28			
	Year 3	0.40				0.07	0.17	0.10		
	Year 4	0.34				0.19	0.29	0.01	0.12	
	Year 5	0.44				0.01	0.09	0.19	0.08	0.2
Job satisfaction	New Graduate	2.72	0.7241	0.0807	0.521					
	Year 1	2.78				0.07				
	Year 2	2.57				0.16	0.22			
	Year 3	2.62				0.11	0.17	0.05		
	Year 4	2.75				0.03	0.03	0.19	0.14	
	Year 5	2.50				0.24	0.31	0.09	0.14	0.28
Career satisfaction	New Graduate	3.18	0.9207	0.0249	0.861					
	Year 1	3.14				0.04				
	Year 2	3.12				0.06	0.02			
	Year 3	3.33				0.16	0.20	0.21		
	Year 4	3.10				0.08	0.05	0.03	0.24	
	Year 5	3.14				0.04	0.00	0.02	0.20	0.05
Work schedule	New Graduate	3.18	0.8306	0.0000	0.670					
	Year 1	3.04				0.15				
	Year 2	2.88				0.33	0.18			
	Year 3	2.85				0.36	0.21	0.02		
	Year 4	3.02				0.17	0.02	0.16	0.19	
	Year 5	2.83				0.38	0.23	0.05	0.03	0.21
Opportunities for advancement	New Graduate	1.71	0.7516	0.0541	0.237					
	Year 1	2.23				**0.57**				
	Year 2	2.08				**0.41**	0.16			
	Year 3	2.07				0.40	0.18	0.01		
	Year 4	2.32				**0.68**	0.11	0.27	0.28	
	Year 5	2.00				0.32	0.25	0.09	0.08	0.36
Independence at work	New Graduate	2.73	0.6491	0.0805	0.410					
	Year 1	3.14				**0.48**				
	Year 2	2.97				0.28	0.19			
	Year 3	2.80				0.08	0.40	0.20		
	Year 4	2.90				0.20	0.28	0.08	0.12	
	Year 5	2.80				0.08	0.40	0.20	0.00	0.12
Professional status	New Graduate	2.38	0.6615	0.0536	0.279					
	Year 1	3.02				**0.76**				
	Year 2	3.06				**0.80**	0.05			
	Year 3	3.05				**0.79**	0.03	0.01		
	Year 4	2.99				**0.72**	0.04	0.08	0.07	
	Year 5	2.85				**0.56**	0.20	0.24	0.23	0.16
Salary	New Graduate	1.87	0.6289	0.2527	0.346					
	Year 1	1.99				0.13				
	Year 2	1.77				0.11	0.24			
	Year 3	1.78				0.10	0.23	0.00		
	Year 4	2.04				0.18	0.05	0.29	0.29	
	Year 5	1.75				0.13	0.26	0.02	0.02	0.31
Educational opportunities	New Graduate	2.73	0.7597	0.0656	0.627					
	Year 1	2.67				0.07				
	Year 2	2.40				0.36	0.30			
	Year 3	2.36				**0.41**	0.34	0.05		
	Year 4	2.43				0.34	0.27	0.03	0.08	
	Year 5	2.21				**0.57**	**0.50**	0.21	0.16	0.24
Appreciation	New Graduate	1.87	0.6453	0.0846	0.021					
	Year 1	2.22				**0.42**				
	Year 2	1.67				0.23	**0.64**			
	Year 3	1.88				0.02	0.40	0.25		
	Year 4	2.11				0.29	0.13	**0.52**	0.27	
	Year 5	1.76				0.13	**0.54**	0.10	0.14	0.41
Immediate supervisor	New Graduate	2.59	0.9221	0.0312	0.368					
	Year 1	2.81				0.23				
	Year 2	2.91				0.32	0.09			
	Year 3	2.58				0.01	0.24	0.33		
	Year 4	2.95				0.37	0.14	0.05	0.38	
	Year 5	2.70				0.11	0.12	0.21	0.12	0.26
Co-workers	New Graduate	3.25	0.6602	0.0043	0.102					
	Year 1	2.97				0.34				
	Year 2	2.87				**0.46**	0.13			
	Year 3	2.83				**0.51**	0.17	0.05		
	Year 4	3.21				0.04	0.30	**0.42**	**0.47**	
	Year 5	2.87				**0.46**	0.12	0.01	0.05	0.42
Work-life balance	New Graduate	2.61	0.7259	0.0355	0.974					
	Year 1	2.52				0.10				
	Year 2	2.41				0.24	0.13			
	Year 3	2.49				0.14	0.04	0.10		
	Year 4	2.56				0.06	0.04	0.18	0.08	
	Year 5	2.50				0.13	0.03	0.11	0.01	0.07
WPV Personal	New Graduate	2.34	0.5668	0.0289	0.238					
	Year 1	1.38				1.25				
	Year 2	1.56				1.02	0.23			
	Year 3	1.66				0.88	0.36	0.14		
	Year 4	1.52				1.07	0.18	0.05	0.18	
	Year 5	1.65				0.89	0.35	0.13	0.01	0.17
WPV Physical	New Graduate	1.83	0.5464	0.000	0.845					
	Year 1	1.33				0.67				
	Year 2	1.47				0.49	0.19			
	Year 3	1.45				0.51	0.16	0.03		
	Year 4	1.42				0.56	0.11	0.07	0.05	
	Year 5	1.49				0.45	0.22	0.03	0.06	0.11
MBI (EE) Total	New Graduate	40.01	199.794	14.151	0.700					
	Year 1	34.78				0.36				
	Year 2	33.17				0.47	0.11			
	Year 3	36.92				0.21	0.15	0.26		
	Year 4	34.27				0.39	0.03	0.08	0.18	
	Year 5	37.20				0.19	0.17	0.28	0.02	0.2

Notes: NG = New Graduate; MSE-Mean Standard Error; Hosp = Hospital; p value = statistical significance; WPV = workplace violence; MBI EE = Maslach Burnout Inventory (Emotional Exhaustion Subscale)

## Discussion

The results of this study highlighted critical challenges faced by ECRNs concerning dissatisfaction with their jobs, workplace violence, burnout, and their intention to leave the profession and their current position. NGs experienced more personal and physical workplace violence than any other year group. They were also the least dissatisfied with their professional status and opportunities for advancement, and most satisfied with their co-workers. First-year ECRNs were the most satisfied with their independence at work, and appreciation, recognition, and rewards. Fifth-year ECRNs were the least dissatisfied with educational opportunities. ECRNs in the private sector had higher levels of job satisfaction, work-life balance, salary, appreciation, recognition, and rewards, educational opportunities, work schedule, and opportunities for advancement than those in the public sector. These findings hold substantial implications for South Africa’s healthcare sector and indicate an urgent need to address these issues to enhance the wellbeing of ECRNs and maintain a viable nursing workforce.

NGs experienced higher levels of personal and physical workplace violence with nurse managers being the perpetrators; this could be due to these nurses having to take up their role as registered nurses after completion of a 12-month community service, putting them at a lower rung on the hierarchy then other health professionals. According to Edmonson and Zelonka [9] stakeholders must actively change the work culture by acknowledging the problem, raising awareness, mitigating contributing factors, and creating and enforcing a strong anti-bullying policy. Furthermore, nurse managers need to adapt to relationship- or people-focused leadership practices that will contribute to improving outcomes for the nursing workforce. These nurse managers will use their emotional skills to understand what individual employees feel during difficult times, thereby building trust and responding to staff [10].

NGs were the least satisfied with their professional status compared to year 1–5 ECRNs. Professional status refers to the recognition and acknowledgement of an individual as skilled and knowledgeable in their field of work. NGs in this study had a minimum of 3 months of working experience, and Duchscher [44] has indicated that NGs experienced “transition shock” as an initial stage of role adaption, which Kramer [45] described as NGs feeling inadequate and unprepared for the working environment. There is vast literature focusing on NGs’ unpreparedness for the workplace, and in their review Masso et al. [46] concluded that there is a need to shift focus to address whether the workplace is ready to support NGs. According to Laschinger et al. [47], ECRNs prefer to work in supportive environments that reward their efforts appropriately and assist with their transition to practice; this was positively experienced by ECRNs in this study, as they were mostly satisfied with their co-workers. Supportive work environments were also found to have a significant positive correlation with job satisfaction and retention [48]. Supportive interventions have been implemented internationally to assist NGs to transition to clinical practice. These include but are not limited to transition to practice programmes, which includes a preceptor, internship, mentorship, and preceptorship [49]. It is important for South African hospitals to create tailored supportive, mentoring and preceptorship programmes to support NGs’ transition, which will improve their confidence and professional status.

First-year ECRNs were the most satisfied with their independence at work, and appreciation, recognition, and rewards. ECRNs in this study work independently after 12 months of community service; hence these first-year ECRNs felt satisfied with their independence at work and at being recognised as registered nurses in the hospitals.

ECRNs in their fifth year were least dissatisfied with educational opportunities compared to other year groups of ECRNs. ECRNs seek professional development during their early career, while at 5 years into their career they are towards their mid-career level. Price and Reichert [50] found that at the mid-level of their career nurses are more focused on a work culture that provides respect and recognition for their wealth of experience and dedication from their employers.

Job dissatisfaction among ECRNs was higher in the public sector than in the private sector, especially in relation to salaries, educational opportunities, recognition and rewards, and opportunities for advancement. Disparities between the public and private sector stems from historical, economic and structural inequalities, which influenced resources allocation, patient demographics and operations models between the health sectors [53]. Nurses working in the public sector in South Africa and Ethiopia also expressed dissatisfaction with their jobs due to low salaries and limited opportunities for promotion and training, according to studies conducted by Pillay [38] and Ayalew et al. [19]. In support of these study results, Morton et al.’s [51] study conducted in South African private sectors indicated great job satisfaction among ECRNs due to receiving training to perform competently. The job satisfaction disparity between the public and private sector could be due to the well-established training nursing institutions that exist within the private sector. These nursing institutions provide educational and career advancement opportunities that include training and development consultants, clinical facilitators, and preceptors who support qualified staff in the workplace [52]. Furthermore, Kurniawan et al. [53] indicated that preceptor-based training programmes had a positive correlation with job satisfaction of novice nurses.

The comparison between the private and public sector revealed significant disparities in job satisfaction and other work-related factors among ECRNs. In the private sector these nurses reported higher levels of job satisfaction, work-life balance, salary, appreciation, recognition and rewards, educational opportunities, work schedule, and opportunities for advancement compared to those in the public sector. Identifying best practices from the private sector can potentially contribute to improving the overall work experience for ECRNs in the public sector. According to Price and Reichert [50], ECRNs identified healthy working environments as those that invested in continuing professional development opportunities to ensure growth in their practice and provide optimal quality patient care.

## Limitations and strengths

The limitations of the study include its reliance on cross-sectional data, which limits the ability to assert causality between the workplace experience of ECRNs and nurse outcomes. Because the study relied on self-report, paper-based surveys to gather data, it may have limited the narrative view of the participants regarding the phenomenon of work experience and nurse outcomes. Furthermore, the sampling of hospitals in the province was purposively selected, and although it included a large set of participants, it is not a probability sample; therefore, the findings are not generalisable to other provinces of South Africa.

The strength of this study is that it is the first national study conducted on nurse outcomes of ECRNs in South Africa in all nursing disciplines using a cross-sectional survey. Previous national South African studies were conducted on all categories of nurses between the years 2009–2016 and in specific nursing disciplines. This study gave insight into ECRNs across different provinces and private and public health sectors of South Africa. In the interpretation of the results, some limitations should be considered.

## Conclusions

The results of this study emphasised the need for immediate action to address challenges faced by ECRNs. The South African health sector needs to implement strategies to improve the quality of the work life of ECRNs, to prevent increased attrition rates that exacerbate nurse shortages. These can be achieved through establishing collaborative stakeholders’ engagements between the private and public sector to identify best practices in promoting positive nurse outcomes for ECRNs, and implementing tailored support strategies for ECRNs, such as transition to practice programmes with preceptorship and mentorship. A positive work environment and supportive leadership are pivotal in ensuring positive nurse outcomes for ECRNs, and nurse managers should be at the forefront of ensuring implementation of preceptorship programmes and mentorship programmes that will support and assist these nurses in crafting their nursing career path. Further research is needed to assess the effectiveness of these interventions in improving job satisfaction and retention of ECRNs.

## Data Availability

The datasets used and/or analysed during the current study are available from the corresponding author upon request.

## Abbreviations

ANOVA: Analysis of variance
CEO: Chief Executive Officer
ECRNs: Early-career registered nurses
FUNDISA: Forum of University Nursing Deans of South Africa
HLM: Hierarchical linear modelling
HREC: Health Research Ethics Committee
NGs: Nurse graduates
NRF: National Research Foundation
RN4CAST: Registered Nurse Forecast
SARChI: South African Research Chairs
STROBE: Strengthening the reporting of observational studies in epidemiology
USA: United States of America

## References

[1] Labrague LJ, De Los Santos JA. COVID-19 anxiety among front-line nurses: predictive role of organisational support, personal resilience and social support. J Nurs Manag. 2020;28(7):1653–61. 10.1111/jonm.13121. 32770780 10.1111/jonm.13121 PMC7436313

[2] Ulupinar S, Aydogan Y. New graduate nurses’ satisfaction, adaptation and intention to leave in their first year: A descriptive study. J Nurs Manag. 2021;29(6):1830–40. 33639015 10.1111/jonm.13296

[3] National Department of Health. Report on nursing workforce shortage: the nursing cluster perspective. Department of Health: Republic of South Africa, Pretoria; 2024.

[4] Schutz E. Ageing nurses: A crisis on the horizon [Internet]. News24Life; 2021 Oct 7 [cited 2024 Apr 27]. Available from: https://www.news24.com/life/archive/ageing-nurses-a-crisis-on-the-horizon-20211007

[5] Adams R, Ryan T, Wood E. Understanding the factors that affect retention within the mental health nursing workforce: A systematic review and thematic synthesis. Int J Ment Health Nurs. 2021;30(1):1476–97. 10.1111/inm.12904 10.1111/inm.12904 34184394

[6] Yahyaei AA, Hewison A, Efstathiou N, Carrick-Sen D. Nurses’ intention to stay in the work environment in acute health- care: A systematic review. J Res Nurs. 2022;27(4):374–97. 10.1177/17449871221080731. 35832883 10.1177/17449871221080731 PMC9272506

[7] Douglas J, Bourgeois S, Moxham L. Early career registered nurses: how they stay. Collegian. 2020;27(4):437–42. 10.1016/j.colegn.2020.01.004.

[8] Mousa DI. Engaging, empowering, and enabling early career nurses. Indianapolis: Sigma Theta Tau International Honor Society of Nursing; 2022.

[9] Edmonson C, Zelonka C. Our own worst enemies: the nurse bullying epidemic. Nurs Adm Q. 2019;43(3):274–79. 31162347 10.1097/NAQ.0000000000000353 PMC6716575

[10] Cummings GG, MacGregor T, Davey M, Lee H, Wong CA, Lo E, et al. Leadership styles and outcome patterns for the nursing workforce and work environment: A systematic review. Int J Nurs Stud. 2018;47(3). 10.1016/j.ijnurstu.2009.08.006. 10.1016/j.ijnurstu.2009.08.006 19781702

[11] Liu Y, Aungsuroch Y, Yunibhand J. Job satisfaction in nursing: a concept analysis study. Int Nurs Rev. 2016;63(1):84–91. 10.1111/inr.12215. 26492403 10.1111/inr.12215

[12] Ekici D, Cerit K, Mert T. Factors that influence nurses’ work-family conflict, job satisfaction and intention to leave. New Trends Issues Proc Humanit Soc Sci. 2017;4(2):128–38.

[13] Williams CA, Goode CJ, Krsek C, Bednash GD, Lynn MR. Postbaccalaureate nurse residency 1-Year outcomes. J Nurs Adm. 2007;37(7/8):357–65. 17939467 10.1097/01.nna.0000285112.14948.0f

[14] Ackerson K, Stiles K. Value of nurse residency programs in retaining new graduate nurses and their potential effect on the nursing shortage. J Contin Educ Nurs. 2018;49(6):282–88. 10.3928/00220124-20180517-09. 29847687 10.3928/00220124-20180517-09

[15] Coetzee SK, Klopper HC, Ellis SM, Aiken L. A Tale of two systems-nurses practice environment, wellbeing and perceived quality of care in private and public hospitals in South Africa: A questionnaire survey. Int J Nurs Stud. 2013;50:162–73. 10.1016/j.ijnurstu.2012.11.002. 23218020 10.1016/j.ijnurstu.2012.11.002

[16] Sojane JS, Klopper HC, Coetzee SK. Leadership, job satisfaction and intention to leave among registered nurses in the North West and free state provinces of South Africa. Curationis. 2016;39(1):1585. 10.4102/cur.v39i1.1585. 27424333 10.4102/cur.v39i1.1585 PMC6091722

[17] Tshitangano TG. Factors that contribute to public sector nurses’ turnover in Limpopo Province of South Africa. Afr J Prim Health Care Fam Med. 2013;5(1):1–7.

[18] Sandler M. Why are new graduate nurses leaving the profession in their first year of practice and how does this impact on ED nurse staffing? A rapid review of current literature and recommended reading. Can J Emerg Nurs. 2018;41(1):23–4.

[19] Ayalew F, Kibwana S, Shawula S, Misganaw E, Abosse Z, van Roosmalen J, et al. Understanding job satisfaction and motivation among nurses in public health facilities of Ethiopia: a cross-sectional study. BMC Nurs. 2019;18:46. 10.1186/s12912-019-0373-8. 31636508 10.1186/s12912-019-0373-8 PMC6794848

[20] De Oliveira DR, Griep RH, Portela LF, Rotenberg L. Intention to leave profession, psychosocial environment and self-rated health among registered nurses from large hospitals in Brazil: A cross-sectional study. BMC Health Serv Res. 2017;17(1):1–10. 10.1186/s12913-016-1949-6. 28068999 10.1186/s12913-016-1949-6 PMC5223488

[21] Maslach C, Leiter MP. Early predictors of job burnout and engagement. J Appl Psychol. 2008;93(3):498–512. 18457483 10.1037/0021-9010.93.3.498

[22] Kapu AN, Borg CE, Jackson H, Kleinpelle R, Kendall J, Lupear BK, et al. Assessing and addressing practitioner burnout: results from an advanced practice registered nurse health and well-being study. J Am Assoc Nurse Pract. 2019;33(1):38–48. 31702604 10.1097/JXX.0000000000000324

[23] Bourdeanu L, Peral ZQ, De Samper M, Pericak KA, Pericak A. Burnout, workplace factors and intent to leave among hematology/oncology nurse practitioners. J Adv Pract Oncol. 2020;11(2):141–48. 33532113 10.6004/jadpro.2020.11.2.2 PMC7848810

[24] Mills J, Woods C, Harrison H, Chamberlain-Salaun J, Spencer B. Retention of early career registered nurses: the influence of self-concept, practice environment and resilience in the first five years post-graduation. J Res Nurs. 2017;22(5):372–85. 10.1177/1744987117709515.

[25] Jarden RJ, Jarden A, Weiland TJ, Taylor G, Bujalka H, Brockenshire N, Gerdtz MF. New graduate nurse wellbeing, work wellbeing and mental health: A quantitative systematic review. Int J Nurs Stud. 2021;121:103997. 10.1016/j.ijnurstu.2021.103997. 34218048 10.1016/j.ijnurstu.2021.103997

[26] Sexton B, Levine LK, Flores D. An analysis of nurse burnout in the emergency department. J Bus Behav Sci. 2021;33:94–108.

[27] Spence Laschinger HK, Wong C, Read E, Cummings G, Leiter M, Macphee M, et al. Predictors of new graduate nurses’ health over the first 4 years of practice. Nurs Open. 2018;6(2):245–59. 10.1002/nop2.231. 30918676 10.1002/nop2.231 PMC6419115

[28] n der Colff JJ, Rothmann S. Burnout of registered nurses in South Africa. J Nurs Manag. 2014; 22:630–42. 10.1111/j.1365-2834.2012.01467. x. 10.1111/j.1365-2834.2012.01467.x 23405857

[29] Cheung T, Lee PH, Yip PSF. Workplace violence toward physicians and nurses: prevalence and correlates in Macau. Int J Environ Res Public Health. 2017;14:879. 10.3390/ijerph14080879. 28777333 10.3390/ijerph14080879 PMC5580583

[30] Cho H, Pavek K, Steege L. Workplace verbal abuse, nurse-reported quality of care and patient safety outcomes among early-career hospital nurses. J Nurs Manag. 2020;28(6):1250–8. 10.1111/jonm.13071. 32564407 10.1111/jonm.13071

[31] Weaver KB. The effects of horizontal violence and bullying on new nurse retention. J Nurses Prof Dev. 2013;39(3):138–42. 10.1097/NND.0b013e318291c453 23703273

[32] Chang HE, Cho S. Workplace violence and job outcomes of newly licensed nurses. Asian Nurs Res (Korean Soc Nurs Sci). 2026;10(4):217–76. 10.1016/j.anr.2016.09.001. 10.1016/j.anr.2016.09.001 28057313

[33] Kennedy M, Julie H. Nurses’ experiences and Understanding of workplace violence in a trauma and emergency department in South Africa. Health SA Gesondheid. 2013;18(1):1–9.

[34] Khalil D. Health consumers right versus nurses’ right: violence against nurses in Cape Town, South Africa. In: I Needman I, K McKenna, M Kingma, N Oud N, editors Workplace violence in the health sector. Proceedings of the second international conference on workplace violence in the health sector– from awareness to sustainable action. Maastricht, Netherlands: KAVANAH; 2010:191–4.

[35] Pillay L, Coetzee SK, Scheepers N, Ellis SM. The association of workplace violence with personal and work unit demographics, and its impact on nurse outcomes in the KwaZulu-Natal Province. Int J Afr Nurs Sci. 2023;18. 10.1016/j.ijans.2023.100571.

[36] Gebregziabher D, Berhanie E, Berihu H, Belsie A, Teklay G. The relationship between job satisfaction and turnover intention among nurses in axum comprehensive and specialized hospital Tigray, Ethiopia. BMC Nurs. 2020;19:79. 10.1186/s12912-020-00468-x. 32831645 10.1186/s12912-020-00468-0 PMC7437041

[37] Brook J, Aitken L, Webb R, MacLaren J, Salmon D. Characteristics of successful interventions to reduce turnover and increase retention of early career nurses: A systematic review. Int J Nurs Stud. 2019;91:47–59. 10.1016/j.ijnurstu.2018.11.003. 30669077 10.1016/j.ijnurstu.2018.11.003

[38] Pillay R. Work satisfaction of professional nurses in South Africa: A comparative analysis of the public and private sectors. Hum Resour Health. 2009;7:1–10. 10.1186/1478-4491-7-15. 19232120 10.1186/1478-4491-7-15 PMC2650673

[39] South African National Department of Health. Regulations relating to categories of hospitals (Notice R.185). Government Gazette. 2012;35101. Available from: https://ndoh.dhmis.org/owncloud/index.php/s/R5cmdp0gY4Fa43Z

[40] Aiken LH, Sermeus W, Van den Heede K, Sloane DM, Busse R, McKee M, et al. Patient safety, satisfaction, and quality of hospital care: cross sectional surveys of nurses and patients in 12 countries in Europe and the united States. BMJ. 2012;344:e1717. 10.1136/bmj.e1717. 22434089 10.1136/bmj.e1717 PMC3308724

[41] Einarsen S, Notelaers G. Measuring exposure to bullying and harassment at work. Validity, factor structure and psychometric properties of negative acts questionnaire revised. Work Stress. 2009;23(1):24–44.

[42] Habibzadeh F. Data distribution: normal or abnormal? J Korean Med Sci. 2024;39(3):e35. 10.3346/jkms.2024.39.e35. PMID: 38258367; PMCID: PMC10803211. 38258367 10.3346/jkms.2024.39.e35 PMC10803211

[43] Cohen J. Statistical power analysis for the behavioral sciences. 2nd ed. New Jersey: Lawrence Erlbaum Associates; 1988.

[44] Duchscher JB. Transition shock: the initial stage of role adaptation for newly graduate registered nurses. J Adv Nurs. 2009;65(5):1103–113. 19183235 10.1111/j.1365-2648.2008.04898.x

[45] Kramer M. Reality shock: why nurses leave nursing. Am J Nurs. 1974;75:891.

[46] Masso M, Sim J, Halcomb E, Thompson C. Practice readiness of new graduate nurses and factors influencing practice readiness: A scoping review of reviews. Int J Nurs Stud. 2022;129:104208. 10.1016/j.ijnurstu.2022.104208. 35344839 10.1016/j.ijnurstu.2022.104208

[47] Laschinger HKS, Wong CA, Grau AL. The influence of authentic leadership on newly graduated nurses’ experiences of workplace bullying, burnout and retention outcomes: A cross-sectional study. Int J Nurs Stud. 2012;49(10):1266–76. 10.1016/j.ijnurstu.2012.05.012. 22727121 10.1016/j.ijnurstu.2012.05.012

[48] Wei H, Sewell KA, Woody G, Rose MA. The state of the science of nurse work environments in the united States: A systematic review. Int J Nurs Sci. 2018;5(3):287–300. 10.1016/j.ijnss.2018.04.010. 31406839 10.1016/j.ijnss.2018.04.010 PMC6626229

[49] Gautam S, Poudel A, Paudyal K, Prajapati MM. Transition to professional practice: perspectives of new nursing graduates of Nepal. BMC Nurs. 2023;22:273. 10.1186/s12912-023-01418-2. 37596552 10.1186/s12912-023-01418-2 PMC10436385

[50] Price S, Reichert C. The importance of continuing professional development to career satisfaction and patient care: meeting the needs of novice to mid- to late-career nurses throughout their career span. Admin Sci. 2017;7(17):1–13.

[51] Morton D, Bowers C, Wessels L, Koen A, Tobias J. Job satisfaction of registered nurses in a private critical care unit in the Eastern cape: A pilot study. Health SA Gesondheid. 2020;25:1–9. 10.4102/hsag.v25i0.1345. 10.4102/hsag.v25i0.1345 PMC7736666 33354354

[52] Vasuthevan S, van Zyl A, Nell S, Kotze E. The private sector contribution to nursing education (1990–2010). Trends Nurs. 2013;1(1). 10.14804/1-1-34.

[53] Kurniawan MH, Hariyati RTS, Afifah E. The relationship between caring preceptor, self-efficacy, job satisfaction, and new nurse performance. Enferm Clin. 2019;29:464–70. 10.1016/j.enfcli.2019.04.

[54] World Medical Association. WMA Declaration of Helsinki– Ethical Principles for Medical Research Involving Human Participants. In. 2013. 10.1001/jama.2024.21972 39425955

